# Expression of mRNA for molecules that regulate angiogenesis, endothelial cell survival, and vascular permeability is altered in endothelial cells isolated from db/db mouse hearts

**DOI:** 10.1007/s00418-024-02327-4

**Published:** 2024-09-24

**Authors:** Krzysztof Bartkowiak, Mateusz Bartkowiak, Ewa Jankowska-Steifer, Anna Ratajska, Elżbieta Czarnowska, Marek Kujawa, Olga Aniołek, Justyna Niderla-Bielińska

**Affiliations:** 1 Histology and Embryology Department, Medical University of Warsaw, Chalubinskiego 5 Str, 02-004 Warsaw, Poland; 2 Department of General, Transplant and Liver Surgery, Medical University of Warsaw, Warsaw, Poland; 3 Department of Pathology, Medical University of Warsaw, Warsaw, Poland; 4 Department of Histology and Embryology, Faculty of Medicine, Lazarski University, Warsaw, Poland

**Keywords:** Angiogenesis, Heart failure, VEGF, VEGFR, Endothelial cell, Blood vessels

## Abstract

Metabolic syndrome (MetS) is a condition that includes symptoms, such as obesity, hyperglycemia, and hypertension, which elevate cardiovascular risk. An impaired angiogenic response of endothelial cells (ECs) in heart and peripheral organs has been proposed in MetS, but the mechanisms of this phenomenon have not been thoroughly explored. Results obtained from evaluating the whole myocardium are inconsistent, since different types of cells react differently to MetS environment and a variety of molecular pathways are involved in the angiogenic response. Therefore, the aim of this paper was to study one selected pathway—the VEGF/VEGFR pathway, which regulates the angiogenic response and microvascular permeability in ECs isolated from db/db mouse hearts. The expression of mRNAs for VEGF/VEGFR axis proteins was assessed with RT-PCR in ECs isolated from control and db/db mouse myocardium. The density of CD31-, VEGFR2-, and VE-cadherin-positive cells was examined with confocal microscopy, and the ultrastructure of ECs was analyzed with transmission electron microscopy. The aortic ring assay was used to assess the capacity of ECs to respond to angiogenic stimuli. Our results showed a decreased number of microvessels, diminished expression of VE-cadherin and VEGFR2 and widened gaps between the ECs of microcapillaries. The aortic ring assay showed a diminished number of sprouts in db/db mice. These results may indicate that ECs in MetS enhance the production of mRNA for VEGF/VRGFR axis proteins, yet sprout formation and vascular barrier maintenance are limited. These novel data may provide a foundation for further studies on ECs dysfunction in MetS.

## Introduction

Obesity is a pandemic of modern times, since one-third of the human population is obese, if obesity is determined by a body mass index (BMI) of ≥ 30. Obesity is a major health care problem, as treatment of its complications is costly, and the number of obese people is increasing constantly (Meldrum et al. 2017). Obese people suffer from many diseases and exhibit a 60% higher risk of death (Afshin et al. 2017). Obesity, especially of the abdominal type, increases also the risk of cardiovascular disease and cardiovascular events by a factor of five (Ortega et al. 2016). Furthermore, obese people very often develop hypertension, hyperglycemia, and dyslipidemia. These symptoms and conditions are collectively referred to as metabolic syndrome (MetS) (Sherling et al. 2017).

MetS environment may lead to accelerated atherosclerosis, fatty liver disease, kidney insufficiency, and/or heart failure (Fahed et al. 2022). Typically, MetS leads to the development of heart failure with preserved ejection fraction (HFpEF) (Purwowiyoto and Prawara 2021). Various detrimental changes within the heart are observed in HFpEF. These changes include cardiomyocyte hypertrophy, reactive oxygen species (ROS) formation, coronary microvessel dysfunction associated with edema, which leads to fibrosis, and an increased ventricular stiffness (Mohammed et al. 2015). Endothelial cell (EC) metabolism dysregulation may impair the angiogenic response and trigger cell death (Salvatore et al. 2022). This results in insufficient nutrition supply and hypoxia, with the latter being a key factor in the development of cardiac inflammation and progression of heart failure (Saltiel and Olefsky 2017; Simmonds et al. 2020).

Impaired angiogenesis in MetS hearts has been well documented, but the causative mechanisms have not been precisely explored (Mazidi et al. 2017; Cheng et al. 2023; Bartkowiak et al. 2024). Formation of new blood vessels is regulated mainly via proangiogenic factors, such as VEGF or FGF, and by their receptors (VEGFR and FGFR), as well as by costimulatory molecules, such as neuropilins (Karaman et al. 2018; Peach et al. 2018). Receptor activation triggers intracellular downstream signaling pathways, among which the Akt and MAP pathways are crucial for events necessary for an angiogenic response, such as cell proliferation, cell migration, and sprout formation. Regulation of vascular permeability is also dependent on VEGF receptors but proceeds via a separate signaling pathway, namely via AKT and/or SRC activation (Simons et al. 2016). EC dysfunction in MetS has been linked to oxidative stress and metabolic deregulation of ECs, which in turn affect vascular tone, permeability, angiogenesis, and surface molecule expression, and may subsequently trigger leukocyte adhesion, inflammation, and EC death (Liu et al. 2023; Veitch et al. 2022).

Apart from VEGFs and their receptors, there are many other proangiogenic factors, notably leptin, which is able to exert effects similar to those of VEGF-A in vitro (Tahergorabi and Khazaei 2015). Leptin is an adipokine produced mostly within white adipose tissue, and it exerts both central and peripheral effects. This adipokine not only regulates energy homeostasis via central appetite regulation, but is also involved in reproduction, homeostasis, and immune function, via leptin receptors (LEPRs), which are found on various types of cells (Wauman and Tavernier 2011). MetS is associated with elevated serum leptin levels, which generates hypothalamic resistance to leptin signaling. There is also some data on peripheral leptin resistance in the heart owing to reduced LEPR expression or inactive LEPRs (Ren et al. 2008; Leifheit-Nestler et al. 2013). Leptin signaling malfunction is also observed in the ECs of obese individuals. It occurs either owing to LEPR downregulation or alterations in receptor binding sites within ECs. Abnormal leptin signaling may lead to an inflammatory response and atherosclerosis, but the mechanism in which the altered leptin signaling impacts cardiac microvessel angiogenesis has not been fully understood (Raman and Khanal 2021).

Our data show significant ultrastructural changes within the microvasculature of cardiac muscle obtained from db/db mice. These changes include increased gaps between ECs and a decreased vesicular index. Additionally, the number of vessels is decreased in db/db mouse myocardium. This may be a result of abnormalities in main signalling pathways involving VEGFR2 and its downstream molecules. Cardiac tissue consists of many different types of cells, among which cardiomyocytes predominate. ECs respond differently to an unfavorable MetS environment. Therefore, the aim of this study was to assess the expression of proangiogenic factors and their receptors in the whole myocardium of db/db mice and in isolated cardiac ECs; we also evaluated intracellular signaling pathways that regulate new blood vessel formation and vascular permeability. Db/db mice are considered to be an animal model of MetS (Fellmann et al. 2013; Alex et al. 2018).

## Methods

### Animals

BKS.Cg-Dock7^m^+/+Lepr^db^/J male mice (db/db; strain no. 000642, breeding location: Italy, order no. BKDSIMA09SS) and C57BL/6J (strain no. 000664, breeding location: Germany, order no. B6JSIMA09W) control mice were used for our experiments. All animal experiments had been approved by the First Local Committee for Ethics in Animal Experiments in Warsaw (at the University of Warsaw, Poland, license no. 140/2016). Nine-week-old male mice were purchased from Charles River Laboratory (251 Ballardvale Str., Wilmington, MA 01887, USA) and kept under specific pathogen-free conditions, 12/12 h dark/light cycle, at 20–24 °C, with access to water and LabDiet® 5K52 (6% fat) chow (Charles River Laboratory, 251 Ballardvale Str., Wilmington, MA 01887, USA) ad libitum. The animals were sacrificed at the age of 21 weeks by CO_2_ asphyxiation, and their hearts and aortas were isolated for further experiments.

### Cardiac EC isolation by magnetic sorting

The hearts collected from 21-week-old db/db and control mice were cut in half, and rinsed in phosphate buffered saline (PBS). Next, the hearts were cut into pieces and digested with 0.5 mg/mL collagenase type II (Sigma-Aldrich, 3300 Str., St. Louis, MO 63118, USA) on a magnetic stirrer at 37 °C for 45 min. To obtain single cell suspensions, the digested tissue was pipetted and filtered through 40-µM nylon filters (Corning, 60 O’Connor Rd., Fairport, NY 14450, USA), and the cells were then washed with PBS and sorted with a magnetic-activated cell sorting system (MACS, Miltenyi Biotec, Inc., 2303 Lindbergh Str., Auburn, CA 95602, USA), according to the manufacturer’s instructions. In brief, the cells were first incubated with primary rat antibodies against CD31 (cat. no 550274, BD Pharmingen Inc., 10975 Torreyana Rd., San Diego, CA 92121, USA) and CD45 (cat no. 550539, 10975 Torreyana Road San Diego, CA 92121 United States). Next, they were incubated with secondary anti-rat IgG antibodies conjugated with microbeads (cat no. 1300-048-501, Miltenyi Biotec, Inc., 2303 Lindbergh Str., Auburn, CA 95602, USA). After being labeled with the antibodies, the cells were separated with MACS Columns and Separators (Miltenyi Biotec, Inc., 2303 Lindbergh Str., Auburn, CA 95602, USA). First, the cells were subdivided into two populations: CD45-positive and CD45-negative. Subsequently, CD31-positive cells were isolated from the latter population. The obtained cells were immediately lysed with lysis buffer. Cells from one heart were collected for reverse transcription polymerase chain reaction (RT-PCR).

### Total RNA isolation, reverse transcription (RT), and Real-Time PCR

In total, 30 mg tissue samples taken from mouse myocardia were transferred to lysis buffer and homogenized. Isolated cells were washed with ice-cold PBS and suspended in lysis buffer. Total RNA was isolated with a NucleoSpin ®RNA II kit (Macherey–Nagel, Valencienner Str. 11, 52355 Düren, Germany) according to the manufacturer’s protocol. The concentration and purity of RNA were estimated with a NanoDrop spectrophotometer (NanoDrop Technologies LLC, 3411 Silverside Rd., Bancroft Building Wilmington, DE 19810, USA). Then, 500 ng of total RNA was reverse transcribed with a High-Capacity RNA-to-cDNA kit (Thermo Fisher Scientific, 168 Third Ave., Waltham, MA 02451, USA), according to the manufacturer’s protocol. cDNA was stored at −20 °C for further experiments. Gene expression was measured with relative quantitation (RQ) with a comparative CT assay. Real-Time PCR was performed with Abi Prism 7500 (Thermo Fisher Scientific, 168 Third Ave., Waltham, MA 02451, USA) in 96-well optical plates. Each sample was run in triplicate, mouse glyceraldehyde 3-phosphate dehydrogenase (GAPDH; Mm99999915_g1) was used as an endogenous control. TaqMan gene expression assays (Thermo Fisher Scientific, MA, USA) were used to measure mRNA for selected genes: PLCγ Mm00549418_m1, cat. no. 4448892; PKCβ2 Mm00435749_m1, cat. no. 4448892; RAF1 Mm00466513_m1, cat. no. 4448892; MEK Mm00488759_m1, cat. no. 4448892; TSAd Mm00451631_m1, cat. no. 4448892; VEGF-A Mm00437306_m1, cat. no. 4453320, VE-cadherin Mm00486938_m1, cat. no. 4453320; eNOS Mm00435217_m1, cat. no. 4453320; VEGF-B Mm00442102_m1, cat. no. 4453320; VEGFR1 (Flt1) Mm00438980_m1, cat. no. 4453320, ERK Mm00442479_m1, cat. no. 4453320; Ki67, cat. no. Mm01278617_m1, cat. no. 4453320; FOXO1 Mm00490671_m1, cat. no. 4453320; FOXO3a Mm01185722_m1, cat. no. 4453320; PTEN Mm00477208_m1, cat. no. 4453320; PIK3ca Mm00435673_m1, cat. no. 4448892; SRC Mm00436785_m1 4448892; FAK Mm00433209_m1, cat. no. 4448892 PXN; Mm00448533_m1, cat. no. 4448892; VEGFR2 Mm01222421_m1, cat. no. 4453320; AKT1, Mm01331626_m1, cat. no. 4453320. All primer and probe sets were purchased from Thermo Fisher Scientific (168 Third Ave., Waltham, MA 02451, USA). The reactions were run with TaqMan Universal Master Mix (Thermo Fisher Scientific, 168 Third Ave., Waltham, MA 02451, USA), primer sets, a MGB probe, and a cDNA template (5 ng per reaction) in the universal thermal conditions 10 min at 95 °C and 40 cycles of 15 s at 95 °C and 1 min at 60 °C). The data were analyzed with sequence detection software version 1.4 (Thermo Fisher Scientific, 168 Third Ave., Waltham, MA 02451, USA).

### Confocal microscopic evaluation of cardiac blood microvessels

Myocardial 10 μm-thick cryosections were fixed in buffered 4% paraformaldehyde solution, and immunostained with primary antibodies in various combinations, followed by incubation with secondary antibodies, according to published protocols (Flaht-Zabost et al. 2014). Primary antibodies used were: anti-CD31 (cat no. 550274, BD Pharmingen Inc., 10975 Torreyana Rd., San Diego, CA 92121, USA), final dilution 1:100; anti-VE-cadherin (cat no. AF1002, R&D Systems, Inc., 614 McKinley Place NE, MN 55413, USA), final dilution 1:25; anti-VEGFR2 (cat no. ab51873, Abcam Limited, Discovery Drive, Cambridge Biomedical Campus, Cambridge, CB2 0AX, UK), final dilution 1:25, anti-alfa-SMA (cat no. ab21027, Abcam Limited, Discovery Drive, Cambridge Biomedical Campus, Cambridge, CB2 0AX, UK), final dilution 1:100; and anti-Lyve-1 (cat no. 11-034, Angiobio, Insight Biotechnology Limited PO Box 520. Wembley Middlesex HA9 7YN, UK), final dilution 1:300. Secondary antibodies were: donkey anti-rat immunoglobulin G (IgG) Alexa Fluor 647-conjugated, final dilution 1:500 (cat no. 712-605-153, Jackson ImmunoResearch, 872 W Baltimore Pike, West Grove, PA 19390, USA); donkey anti-goat IgG Fluorescein isothiocyanate (FITC)-conjugated (cat no. 705-095-147, Jackson ImmunoResearch, 872 W Baltimore Pike, West Grove, PA 19390, USA), final dilution 1:200; and donkey anti-rabbit IgG CyTM3-conugated, (cat no. 711-165-152, Jackson ImmunoResearch, 872 W Baltimore Pike, West Grove, PA 19390, USA), final dilution 1:800. Cell nuclei were counterstained with Hoechst (Sigma-Aldrich, 3300 Str., St. Louis, MO 63118, USA), according to the manufacturer’s protocol. Specimens were mounted in fluorescence mounting medium (Dako, Produktionsvej 42, 2600 Glostrup, Denmark). Sections were viewed under confocal microscopes: Olympus FV 1000 (with oil immersion 20×/0.80 objective, images were analyzed with FV1000 Operations Software ver 4.2; Olympus, 2951 Ishikawa-machi, Hachioji-shi, Tokyo 192-8507, Japan), Leica TCS SP5 (with HCX PL APO L 20 × 1.0 water immersion objective, images were analyzed with LasAF Lite 3.3 software; Leica Microsystems GmbH. Ernst-Leitz-Strasse 17-37. 35578 Wetzlar Germany) or Zeiss LSM 780/Elyra PS.1 (with Plan-Apochromat 20×/0,8 objective, images were analyzed with ZEN 2.3; Carl Zeiss, Carl-Zeiss-Straße 22, 73447 Oberkochen, Germany). At least three independent immunostainings were performed on separate tissue sections. Myocardial sections were stained with each respective primary antibody and were used as positive controls. Separate sections without primary antibody treatment were used as negative controls. Photomicrographs show representative cross sections of the cardiac muscle. Microvessel density was evaluated only in regions where cardiomyocytes were transversely cut and at least six regions were randomly selected for calculations. Microvessel density has been presented as the number of CD31^+^/Lyve-1^−^ vessels per 1 mm^2^ of myocardial tissue sections. The density of VE-cadherin and VEGFR2 signal was calculated in relation to CD31^+^/Lyve-1^−^ vessel density.

### Ultrastructure and morphometric analysis of cardiac microvessels

Karnovsky-fixed (half-strength of Karnovsky fixative contains 2.5% glutaraldehyde and 2% paraformaldehyde in 0.1 M of phosphate buffer, pH 7.4) small tissue specimens from mouse hearts were postfixed in 1% buffered osmium tetroxide and processed to Epon embedding (Polysciences, Inc. 400 Valley Road. Warrington, PA 18976, USA), according to a published protocol (Dobrzynska et al. 2015). Ultrathin sections were cut, contrasted with uranyl acetate and lead citrate, and examined by transmission electron microscopy (TEM) (Jeol JEM 1011; Jeol Ltd., 3-1-2 Musashino, Akishima, Tokyo 196-8558, Japan). Dimensions of gaps in areas of intercellular junctions between blood endothelial cells of cardiac microvessel and the number of cytoplasmic vesicles per 1 µm of cellular membrane length (specified here as vesicular indexes) were assessed and measured with a TEM morphometric program (iTEM Olympus, 2951 Ishikawa-machi, Hachioji-shi, Tokyo 192–8507, Japan).

### Aortic ring assay

Collagen-coated culture chambers (Nunc™ Lab-Tek™ II Chamber Slide™ System, Thermo Fisher Scientific, 168 Third Ave., Waltham, MA 02451, USA) were prepared 2 h prior to aorta isolation; 450 μl of collagen solution (STEMCELL Technologies, 1618 Station Str. Vancouver, BC, V6A 1B6, Canada) was mixed on ice with 800 μl of ECM medium supplemented with 1% fetal calf serum (FCS), 1% antibiotic/antimycotic, EC growth supplement of 0.004 ml/ml, EGF of 0.1 ng/ml, bFGF of 1 ng/ml, heparin of 90 μg/ml, and hydrocortisone of 1 μg/ml (cat No. C-22110, PromoCell GmbH, Sickingenstr. 63/65, 69126 Heidelberg, Germany) and immediately transferred to the culture-chamber slide. After 1 h in a 37 °C incubator, the collagen was washed and soaked with ECM medium, supplemented as above. Aortas were isolated from the mice, cleared of adipose tissue, and cut into approximately 1 mm thick rings. Before the aorta rings were placed on the collagen, the culture medium was carefully removed to enable contact with the collagen surface. A medium enriched with mouse VEGF-A_164_ (50 ng/ml, from R&D Systems, Inc., 614 McKinley Place NE, MN 55413, USA) was added to the aorta culture after 18 h. The medium was changed every other day. Cultures were examined under a contrast-phase microscope every other day. After 8 days, the explants were fixed for immunostaining or dissected from the collagen, and remaining sprouts were lysed for RNA isolation.

### Whole-mount immunostaining of aortic sprouts

Cultures of aortic rings were fixed with buffered 4% paraformaldehyde overnight at 4 °C, washed with PBS, and incubated with 1% bovine serum albumin (BSA), 0.1% TritonX-100, and 0.1 M of glycine in PBS for 30 min. After thorough rinsing with PBS, nonspecific background staining was blocked with 5% donkey serum (Jackson ImmunoResearch, 872 W Baltimore Pike, West Grove, PA 19390, USA). Subsequently, specimens were incubated overnight at 4 °C with a primary antibody: anti-CD31 (cat no. 550274, BD Pharmingen Inc., 10975 Torreyana Rd., San Diego, CA 92121, USA) diluted in PBS/1% BSA 1:100. After three washes in PBS, specimens were incubated for 3 h with donkey secondary antibodies diluted in PBS/1% BSA conjugated with a fluorescent particle (donkey anti-rat IgG AlexaFluor® 647, dilution 1:500, cat No. 712-605-153, Jackson ImmunoResearch, 872 W Baltimore Pike, West Grove, PA 19390, USA), washed as above, and cell nuclei were counterstained with Hoechst (Sigma-Aldrich, 3300 Str., St. Louis, MO 63118, USA), according to the manufacturer’s protocol. Specimens were mounted in fluorescence mounting medium (Dako, Produktionsvej 42, 2600 Glostrup, Denmark) and viewed under a Leica confocal microscope, type TCS SP5, with HCX PL APO L 20 × 1.0 water immersion objective. Images were analyzed with LasAF Lite 3.3 software (Leica Microsystems GmbH. Ernst-Leitz-Strasse 17-37, 35578 Wetzlar, Germany). During analysis, the number of sprouts per explant was calculated.

### Statistical analysis

Data were analyzed with GraphPad Prism 9 (GraphPad Software, 2365 Northside Dr., Ste. 560, San Diego, CA 92108, USA). The normality of distribution was assessed by the Shapiro–Wilk test, then the *t*-test or the Mann–Whitney test was applied. Results were considered statistically significant at a *p*-value of ≤ 0.05.

## Results

### In db/db mice the density of microvessels is reducted, and the integrity of the microvascular barrier is compromised

**Fig. 1:**
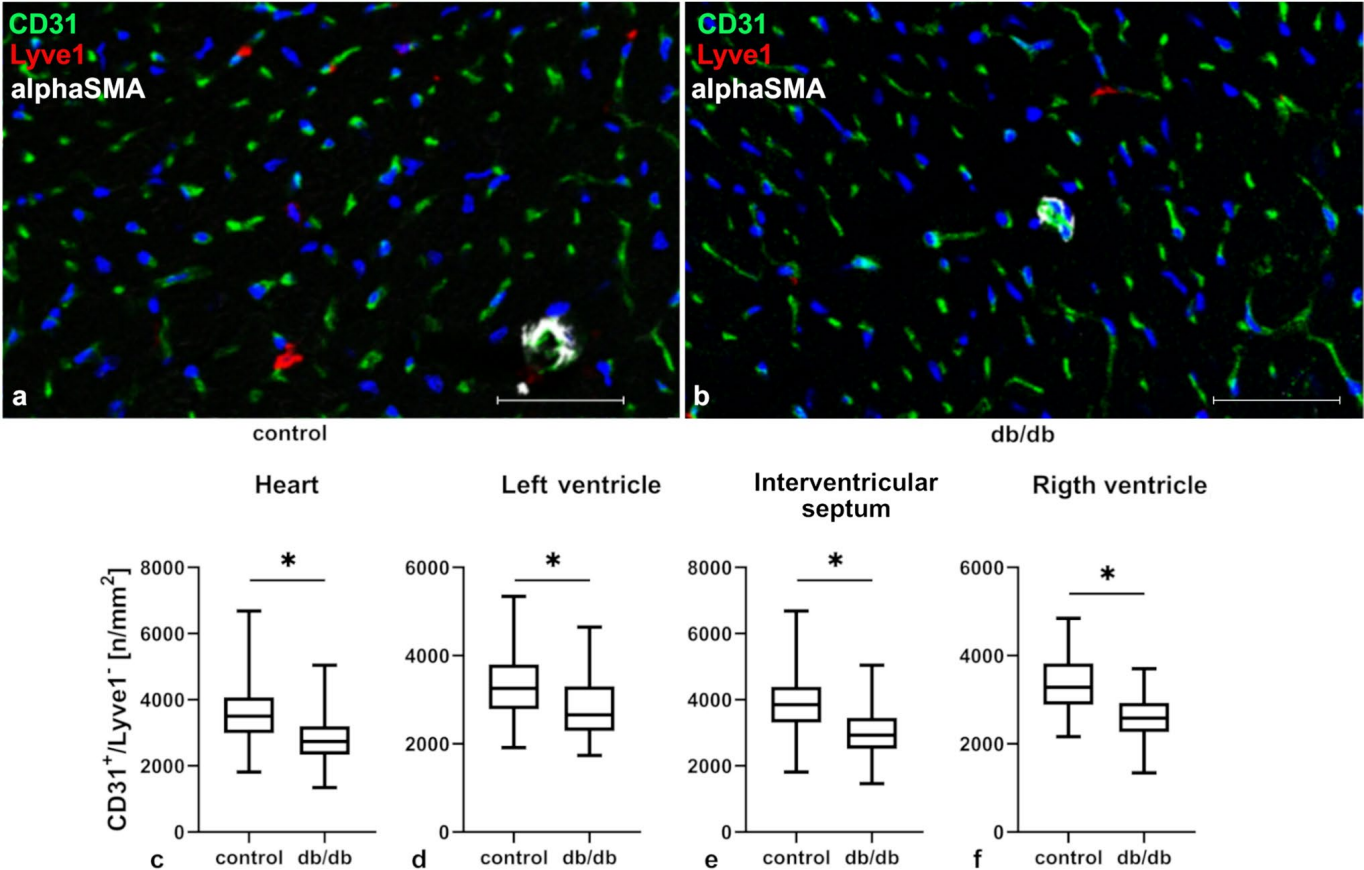
Density of microvessels in cardiac tissue calculated as a number of CD31^+^/Lyve-1^−^ cells per one square mm of tissue in control and db/db mice. Panels **a**, **b** show confocal analysis of CD31^+^/Lyve-1^−^ cells; panels **c–f** show density of CD31^+^/Lyve-1^−^ cells per one square mm of tissue in various parts of the heart. Graphs **c–f** show measurements from three mouse hearts per group; at least six randomly selected regions of interest were analyzed for each location (*n* = 18); only transverse sections of tissue were included into calculations. The normality of the distribution was assessed by the Shapiro–Wilk test. The *t*-test or the Mann–Whitney test were applied depending on data distribution. Results were considered statistically significant at a *p*-value of ≤ 0.05. *p < 0.05 Error bars show standard deviation of the mean

Microvascular density, calculated as a number of CD31^+^/Lyve-1^−^ cells per one square mm of tissue, was evaluated in the hearts of db/db mice and those of controls (Fig. 1). Since experimental animals showed no more cardiomyocyte hypertrophy than controls (data not shown), the number of vessels was counted per unit of area. In the experimental group statistically significant decrease in CD31^+^/Lyve-1^−^ cells in the whole heart, as well as in separate areas of the heart, e.g., the interventricular septum, left ventricle, and right ventricle, were observed (Fig. 1c–f).

**Fig. 2:**
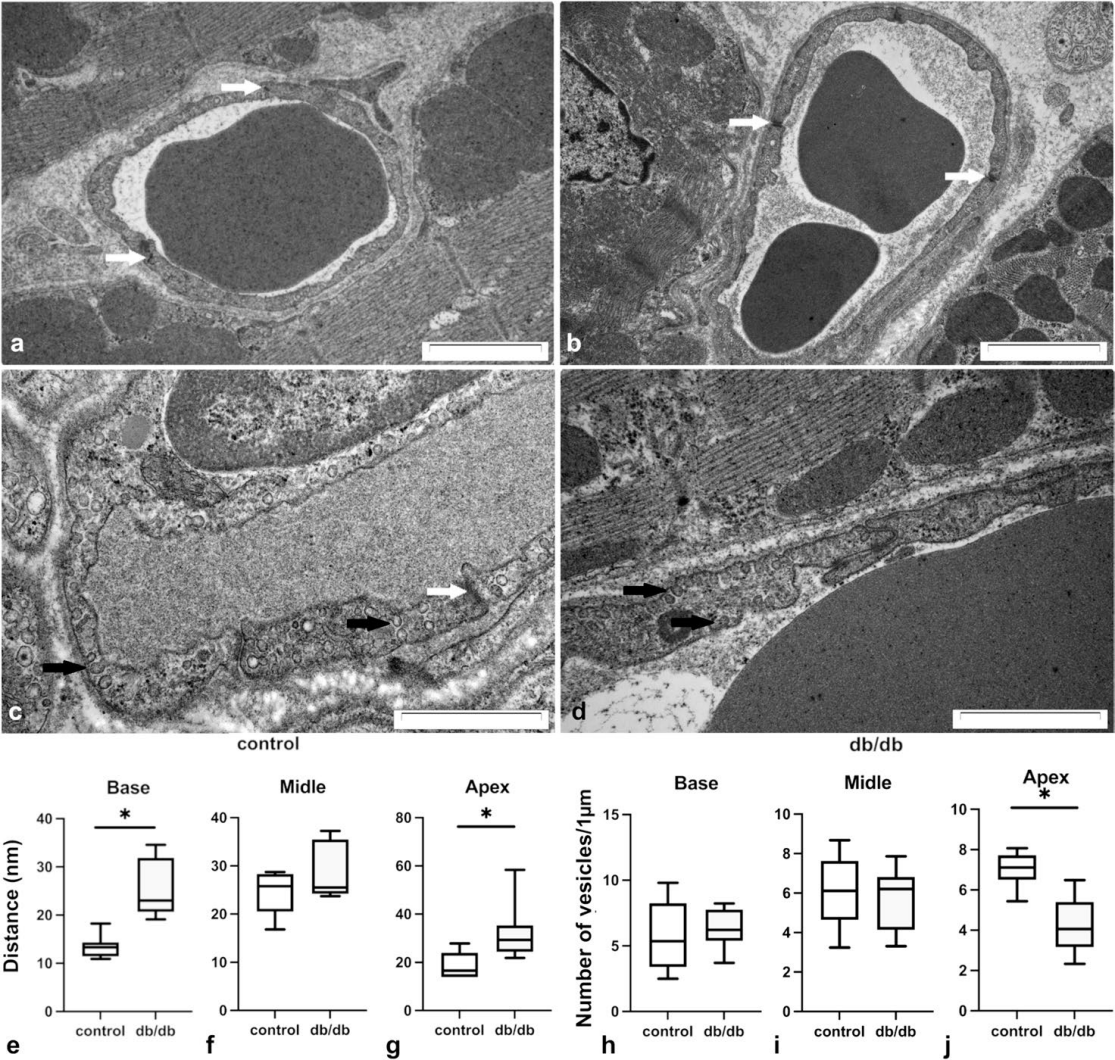
Cardiac blood microcapillary remodeling. TEM details of microcapillaries in a control (**a**, **c**) and db/db (**b**, **d**) mouse. Distances between adjacent ECs (i.e., junctional gaps) and cytoplasmic vesicles in blood capillary ECs are shown in **a–d**, respectively. Junctional gap distances (**e–g**) and the number of vesicles (**h–j**) were calculated in different areas of the heart. Calculations were made on the basis of samples taken from three hearts per group. The number of randomly selected areas for measurements in each group were: (**e–g**) control—base *n* = 8, middle *n* = 6, apex *n* = 6, db/db *n* = 45, db/db—base *n* = 9, middle *n* = 6, apex *n* = 10; (**h–j**) control—base *n* = 10, middle *n* = 10, apex *n* = 10, db/db n = 45, db/db—base *n* = 10, middle *n* = 10, apex *n* = 10, db/db. The normality of distribution was assessed by the Shapiro–Wilk test. The *t*-test or the Mann–Whitney test was used depending on the distribution. The results were considered statistically significant at a *p*-value of ≤ 0.05; *n*—number of regions of interest (randomly selected areas for measurements); white arrows—junctional gaps; black arrows—vesicles in EC cytoplasm. *p < 0.05. Error bars show standard deviation of the mean

Widened junctional gaps between ECs are considered a morphological sign of increased vascular permeability (McDonald et al. 1999). Figure 2a–d illustrates EC morphology in cardiac microcapillaries. The distances between adjacent ECs, i.e., junctional gaps and cytoplasmic vesicles, in blood capillary ECs are shown on Fig. 2a–d. Analysis of gaps with TEM displayed an increased width in db/db in comparison with that in control mice (Fig. 2e–g) at the base and at the apex of the heart. No difference was observed within the middle region of the heart. Furthermore, we observed a decreased vesicular index at the apex of the heart, but not in other regions. This may indicate that active transcellular transport was decreased in the region of the heart apex (Fig. 2h–j).

### The expression of mRNA for crucial receptors and growth factors involved in regulation of angiogenesis and vessel integrity is upregulated only in isolated ECs from db/db mice, but not in the whole myocardium

**Fig. 3:**
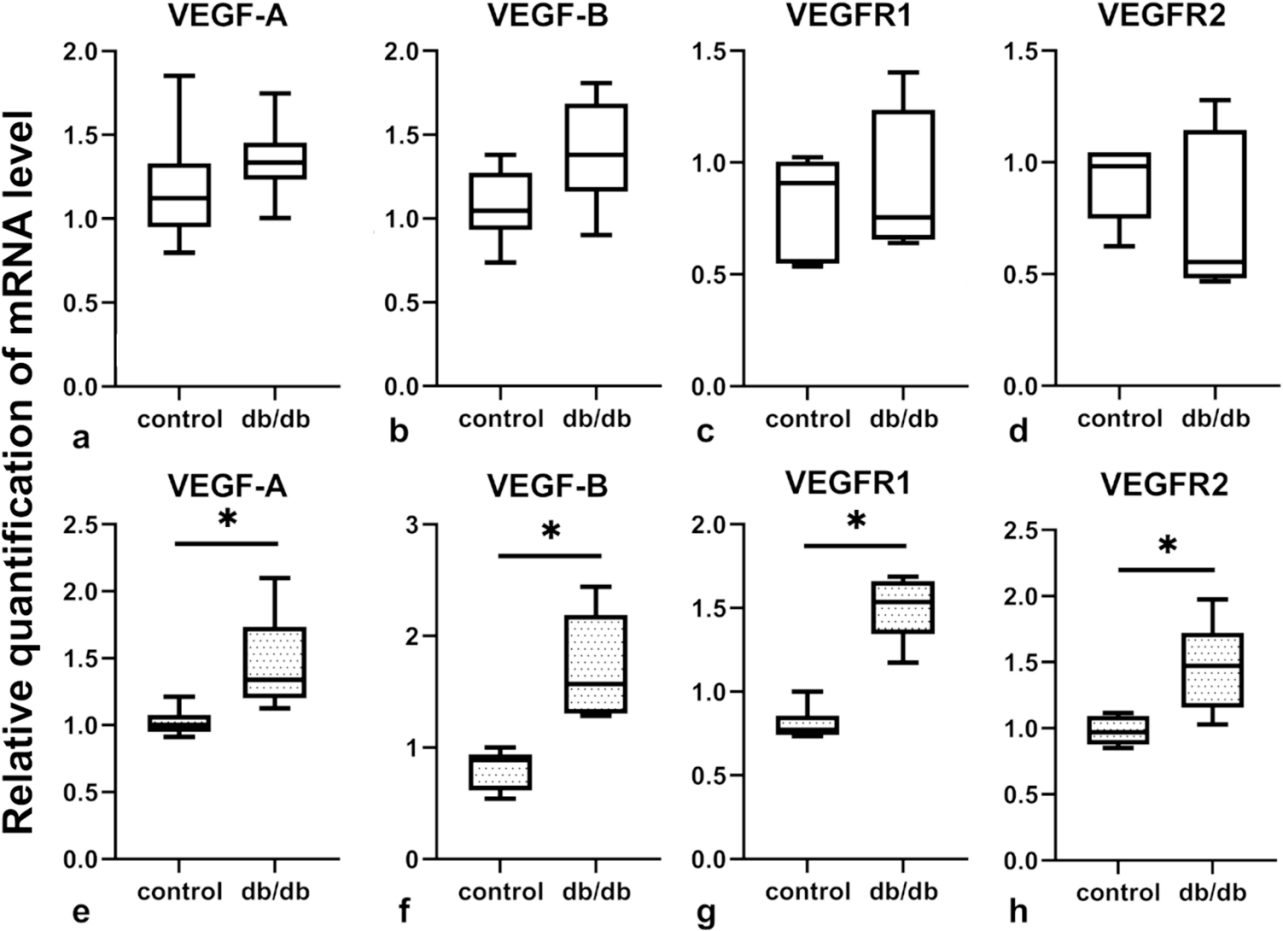
Expression of mRNA for crucial growth factors and receptors involved in the regulation of angiogenesis in control and db/db mouse cardiac tissue and in isolated ECs (*n* = 5 for each group). Panels **a–h** show relative quantification of mRNA for selected molecules involved in angiogenesis regulation in whole cardiac tissue and in isolated ECs, respectively. The normality of the distribution was assessed by the Shapiro–Wilk test. The *t*-test or the Mann–Whitney test was used depending on data distribution. Results were considered statistically significant at a *p*-value of ≤ 0.05; *n*—the number of mice used for whole cardiac tissue mRNA extraction or EC isolation and mRNA extraction. *p < 0.05. Error bars show standard deviation of the mean

**Fig. 4:**
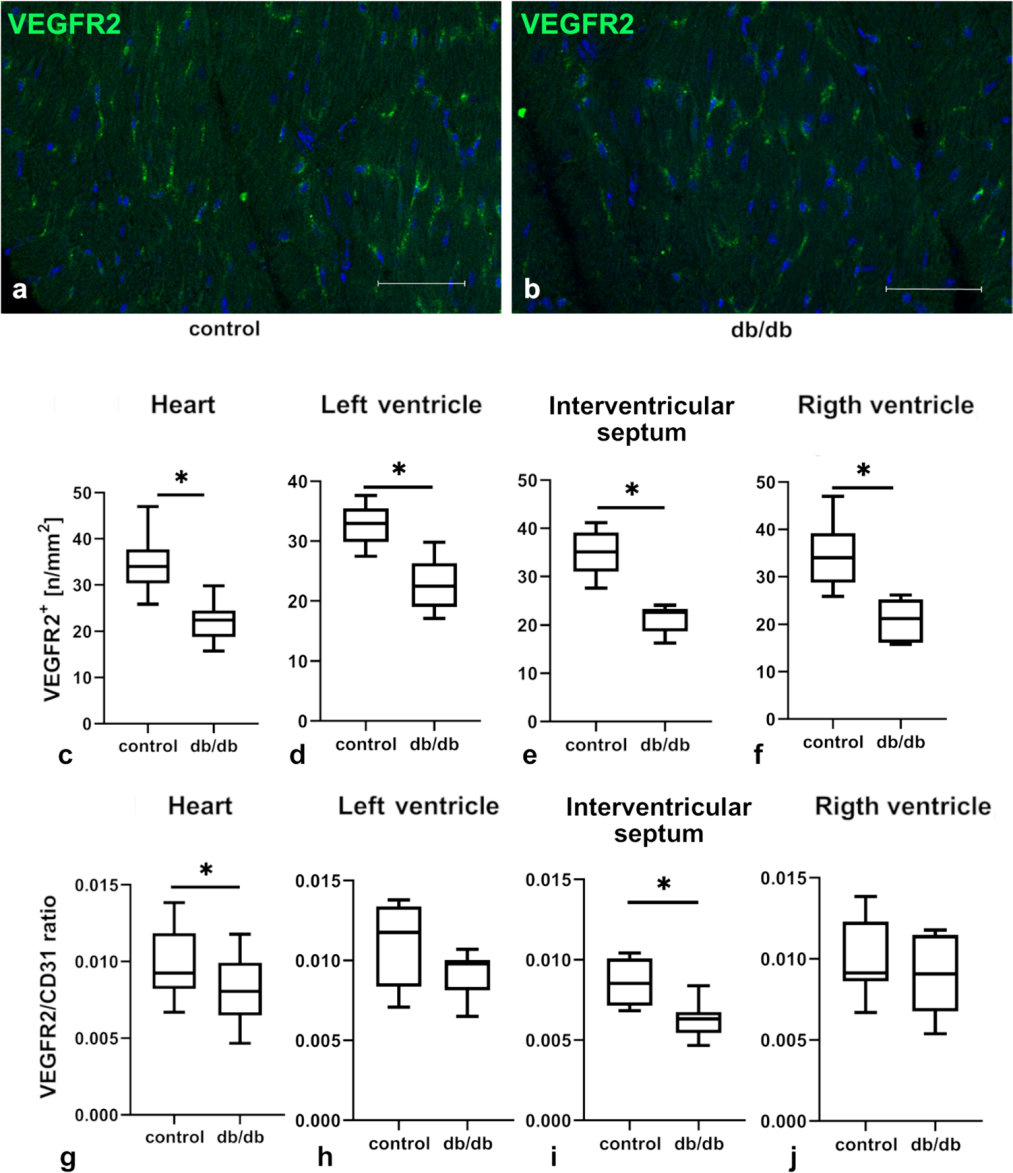
The density of VEGFR2-positive cells in the whole heart and in its regions evaluated via confocal microscopy and measured per 1 mm^2^ of tissue. Panels **a**, **b** show VEGFR2 immunostaining in control and db/db mice, respectively; graphs **c–f** show the number of VEGFR2-positive cells per 1 mm^2^ of tissue in various regions of the heart; graphs **g–j** show the VEGFR2/CD31 ratio in various regions of the heart. **c-f** Show measurements from three mouse hearts per group; at least six randomly selected regions of interest were analyzed for each location (*n* = 18); only transverse sections of tissue were included into calculations. The normality of distribution was assessed by the Shapiro–Wilk test. The *t*-test or the Mann–Whitney test was used depending on data distribution. Results were considered statistically significant at a *p*-value of ≤ 0.05. *p < 0.05. Error bars show standard deviation of the mean

To further analyze the possible mechanism leading to vascular rarefaction and leakiness in db/db mice, the expression of VEGF-A, VEGF-B and VEGFR1, and VEGFR2 in whole cardiac muscle and in isolated ECs were evaluated. There were no statistical differences in the expression of mRNA for VEGF-A, VEGF-B, VEGFR1, or VEGFR2 in the whole cardiac tissue of control and db/db groups (Fig. 3a–d). However, in isolated ECs there was a significantly increased expression of VEGF-A, VEGF-B, and their receptors in the db/db group (Fig. 3e–h). In situ evaluation of VEGFR2 expression showed a significant downregulation of this protein in whole heart, which may reflect the observed decrease in the number of microvessels (Fig. 4a–f). Additionally, VEGFR2/CD31 ratio analysis showed a decreased expression of VEGFR in whole heart and in the interventricular septum, but not in the left or right ventricle (Fig. 4g–j).

### The expression of mRNA for the MAP-signalling pathway is elevated in isolated cardiac ECs from db/db mice

**Fig. 5:**
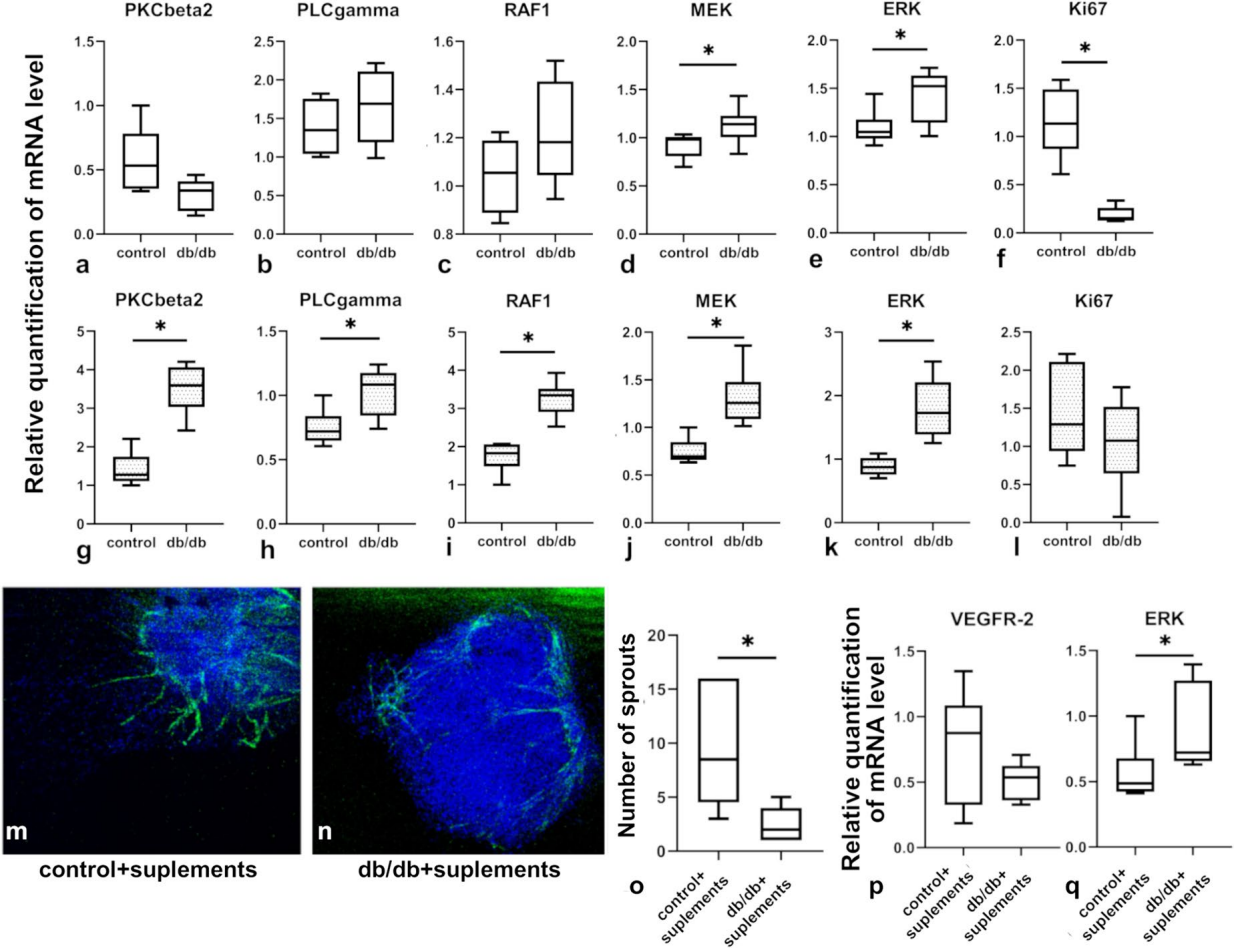
The expression of mRNA for key molecules involved in the MAP-kinase pathway and the ability of ECs to respond to angiogenic stimuli. Panels **a–l** show relative quantification of mRNA for selected molecules involved in the MAP-kinase pathway in the whole cardiac tissue and in isolated ECs, respectively (*n* = 5 for each group). Panels **m**, **n** show a representative result of the aortic ring assay; tubules were stained with anti-CD31 antibodies. Graph (**o**) shows the number of sprouts produced in control- and db/db-mouse-derived aortas under the influence of mouse VEGF-A_165_. Panels **p**, **q** demonstrate relative quantification of mRNA for VGFR2 and ERK in vascular sprouts from the aortic ring assay. Panels **o–q** show measurements from three mouse experiments per group; at least five aortic rings per mouse were analyzed (*n* = 15). The normality of distribution was assessed by the Shapiro–Wilk test. The *t*-test or the Mann–Whitney test was used depending on data distribution. Results were considered statistically significant at a *p*-value of ≤ 0.05. *p < 0.05. Error bars show standard deviation of the mean

To evaluate the possible irregularities in the downstream VEGFR2 signalling cascade, we examined the expression of mRNA for main molecules involved in the MAP-regulated pathway. Only the expression of mRNA for MEK and ERK was significantly increased in the whole heart in db/db mice. Despite this, the expression of mRNA for Ki67 was decreased in db/db hearts in comparison to those in the control group (Fig. 5a–f). In isolated ECs there was upregulation of mRNA for VEGFR2, PKCγβ2, PLCγ, RAF-1, MEK, and ERK (Fig. 5g–l), but the expression of Ki-67 was not affected.

### Sprouting is impaired in the aortic ring assay with db/db-derived aortas stimulated with angiogenic factors

The aortic ring assay was performed with aortas isolated from db/db and control mice. Incubation with proangiogenic VEGF-A_164_ isoform stimulated the formation of sprouts, defined as CD31-positive structures, but the number of sprouts was significantly lower in db/db-derived aortic rings compared with the number of sprouts from control-derived aortic rings (Fig. 5m–o). Furthermore, the mRNA levels for VEGFR2 showed no significant differences between the control and db/db groups, whereas mRNA for ERK1/2 molecules was significantly upregulated in db/db aortas supplemented with proangiogenic factors (Fig. 5p, q).

### The upregulated expression of mRNAs for AKT1 signalling pathways are observed in ECs isolated from cardiac muscle of db/db mice

**Fig. 6:**
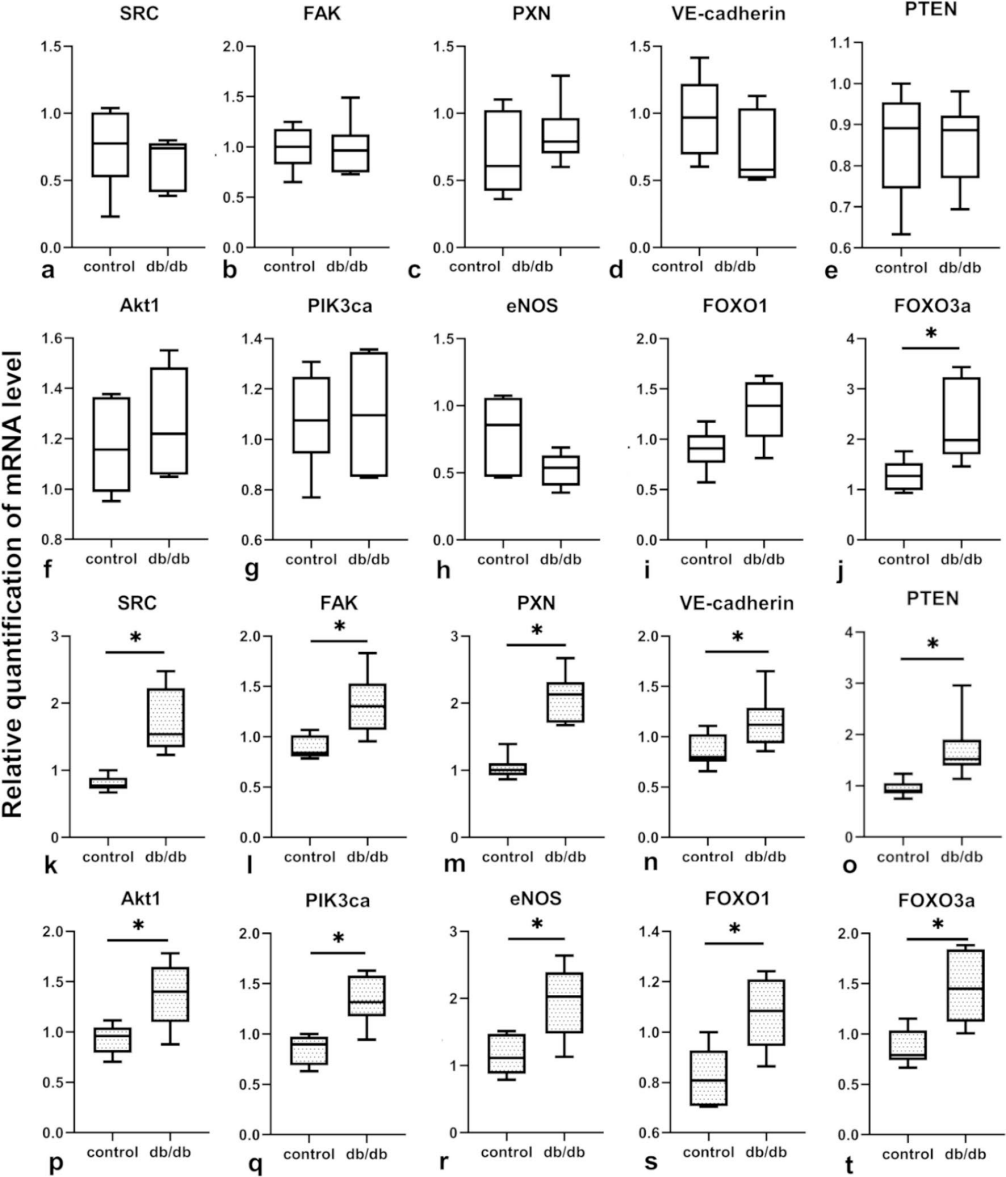
mRNA expression for proteins involved in the AKT signaling pathway in the whole cardiac tissue (**a–j**) and in isolated ECs (**k–t**). Isolated cells and cardiac tissue samples were collected separately from five animals (*n* = 5) in both the control and the db/db group. The normality of distribution was assessed by the Shapiro–Wilk test. The *t*-test or the Mann–Whitney test was used depending on data distribution. Results were considered statistically significant at a *p*-value of ≤ 0.05. *p < 0.05. Error bars show standard deviation of the mean

**Fig. 7:**
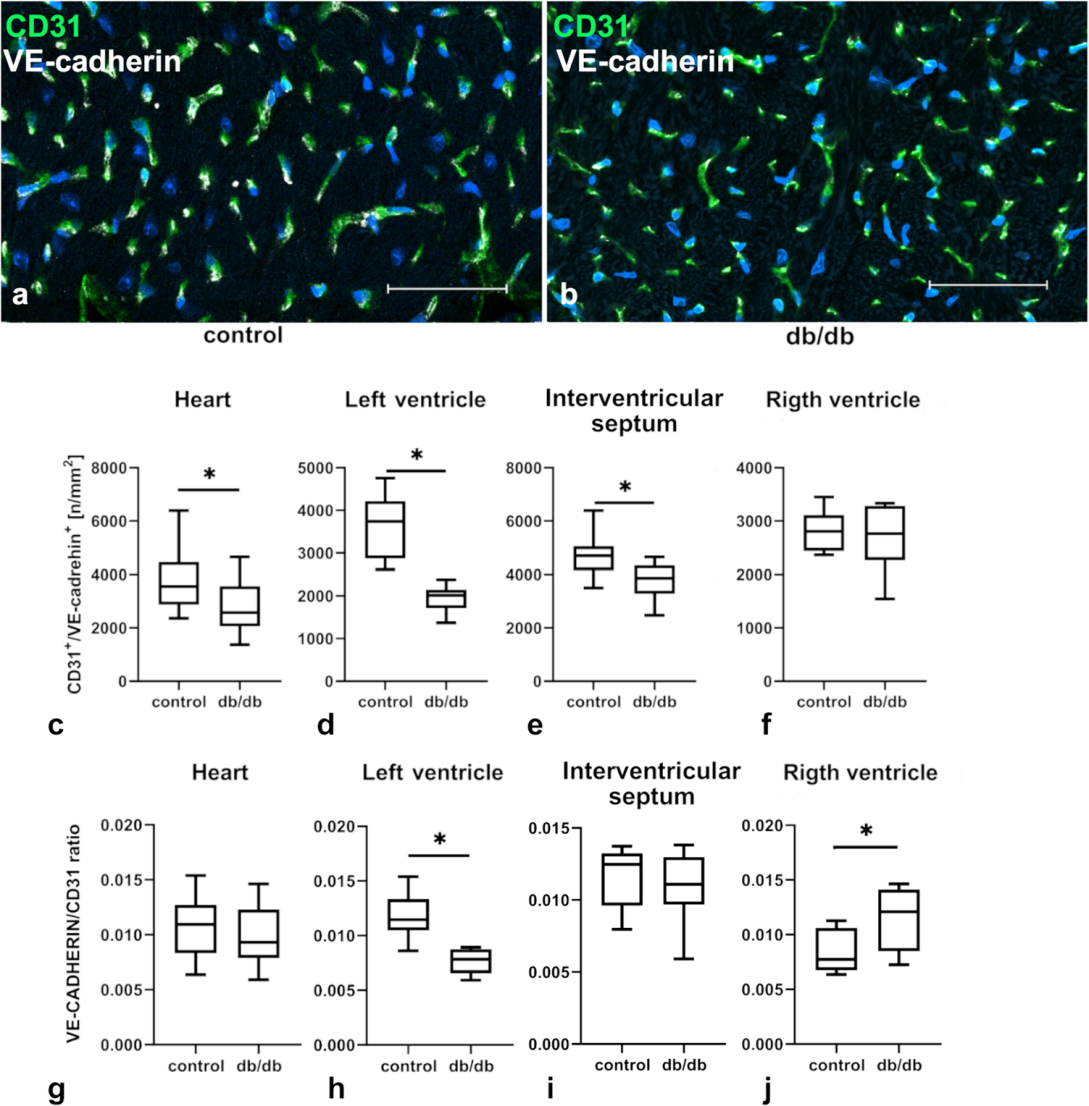
VE-cadherin expression in control and db/db mouse hearts, analyzed with confocal microscopy (**a**, **b**). Graphs **c–f** show density of VE-cadherin and CD31 double-positive cells in the whole heart and in cardiac regions evaluated with confocal microscopy and measured per 1 mm^2^ of tissue. Graphs **g–j** demonstrate the VE-cadherin/CD31 ratio in various regions of the heart. Graphs **c–j** show measurements from three mouse hearts per group; at least six randomly selected regions of interest were analyzed for each location (*n* = 18); only transverse sections of tissue were included into calculations. The normality of distribution was assessed by the Shapiro–Wilk test. The *t*-test or the Mann–Whitney test was used depending on distribution. Results were considered statistically significant at a *p*-value of ≤ 0.05. *p < 0.05. Error bars show standard deviation of the mean

The expression of mRNA for proteins involved in the AKT signalling pathway in whole cardiac tissue was not significantly affected, except for FOXO3a, which was upregulated in db/db cardiac muscle when compared with control cardiac muscle (Fig. 6a–j). On the other hand, all examined mRNAs (SRC, FAK, PXN, VE-cadherin, AKT1, PI3K, eNOS, FOXO1, FOXO3a, and PTEN) were significantly upregulated in isolated cardiac ECs from db/db mice (Fig. 6k–t). The in situ expression of VE-cadherin, evaluated with confocal microscopy, was downregulated in the whole heart and in both ventricles, but not in the interventricular septum, which may be due to a decrease in the number of microvessels in the ventricles of db/db mice (Fig. 7a–f). The VE-cadherin/CD31 ratio showed a decrease of VE-cadherin expression in ECs only in the left ventricle. There was no change in VE-cadherin expression in the whole heart or in the interventricular septum, and an increase in VE-cadherin expression in ECs in the right ventricle (Fig. 7g–j).

## Discussion

### Number and morphology of microvessels in cardiac muscle of db/db mice

MetS symptoms, such as obesity, hyperglycemia, hyperlipidemia, and hypertension, negatively affect the cardiovascular system (Purwowiyoto and Prawara 2021). MetS conditions have been repeatedly reported to alter the expression of molecules involved in angiogenesis and vascular homeostasis, which in turn may contribute to some symptoms of this condition, such as delayed wound healing and impaired collateral growth after cardiac ischemia (Chou et al. 2002; Bartkowiak et al. 2024; Cheng et al. 2023). Moreover, unfavorable changes in cardiac vessel EC metabolism occur before the development of diastolic dysfunction in diabetic individuals (Taqueti et al. 2018). Similarly, microvascular rarefaction and dysfunction in peripheral organs are observed in animal models in prediabetic state, although the data are often contradictory (Veitch et al. 2022; Roy et al. 2022). There are two possibilities discussed—poor angiogenesis and increased apoptosis (Rawal et al. 2017; Niderla-Bielinska et al. 2021; Dham et al. 2021).

Db/db mouse is one of the animal models used for MetS studies (Alex et al. 2018). As a result of a point mutation, leptin receptors in db/db mice become inactivated; therefore, these mice develop central leptin resistance, causing a voracious appetite and subsequent obesity and diabetes. Dysregulation of the central leptin axis is considered a main cause of obesity (Ren 2004), but in humans it is primarily overeating that rapidly increases blood leptin levels, leading to leptin resistance and weight gain (Ren 2004; Berger and Kloting 2021; Schwartz et al. 1996). Therefore, the results obtained in this study cannot be directly translated to patients, although there are some rare congenital LEPR or leptin mutations in humans that can lead to overeating, obesity, and T2DM (Farooqi et al. 2007). It is worth mentioning that there are also mouse models with EC-specific LEPR deficiency, in which the animals develop obesity only when high fat diet is introduced (Gogiraju et al. 2023).

Weight gain and hyperglycemia in db/db mice is associated with alterations in myocardial structure and function, although study results are often contradictory and depend heavily on mouse sex, age, and the specific db/db strain (Alex et al. 2018). In our experiment db/db mice at the of age 21 weeks were already developing severe obesity and hyperglycemia, although heart weight (calculated as heart weight/tibia length) was significantly lower when compared with that in the control group (data shown in (Niderla-Bielinska et al. 2021)). Morphological assessments of cardiac muscle revealed microvascular rarefaction and compromised vascular barrier. Although our first observation was consistent with previously published data (Alex et al. 2018; Rawal et al. 2017; Veitch et al. 2022), vascular barrier integrity in cardiac muscle was never before evaluated in db/db mice. Since we were able to employ only one method of assessing vascular permeability, which was morphological analysis of junctional gaps (McDonald et al. 1999), this observation needs further studies.

### mRNAs for proteins involved in the angiogenic response are altered in ECs isolated from db/db mouse cardiac muscle

VEGFs are crucial proteins involved in regulation of angiogenesis. Elevated levels of VEGF-A in serum of MetS individuals are well documented and positively correlate with the amount of white adipose tissue (Zafar et al. 2018). In contrast, measurements of VEGF and VEGF receptor expression in cardiac tissue show contradictory results. No differences in the expression of VEGF-A, either on mRNA or protein levels were observed in the myocardium of db/db mice compared with controls (Broderick et al. 2012, 2019; Chen et al. 2012). Diabetic rats and diabetic patients show a decrease in VEGF-A and VEGFR2 expression in cardiac tissue (Chou et al. 2002). This may be owing to the fact, that these analyses involved whole cardiac tissue, which is composed of different types of cells that can respond to diabetic environment in different ways (Litviňuková et al. 2020). In our experiment RT-PCR analysis of mRNA expression for VEGF-A and VEGF-B in whole cardiac tissue did not show any changes between db/db and control animals. However, when we performed the same analysis with isolated ECs, we observed a statistically significant increase in mRNA for these factors. Furthermore, the expression of two crucial receptors for VEGFs (VEGFR1 and VEGFR2) also yielded surprising results. In the cardiac tissue there was no change in mRNA expression for these receptors, but when only the ECs are analyzed, increased expression was observed. This is in contrast to previously obtained results, where a reduction of VEGFR1 and VEGFR2 mRNA expression in the whole cardiac tissue in db/db mice was reported (Park et al. 2009). This may be owing to different age of the animals used in these experiments. In literature there is a lack of results showing mRNA expression in isolated ECs (Krüger-Genge et al. 2019). Unfortunately, we were unable to analyze the expression of VEGFs and their receptors on the protein levels owing to the small amount of the ECs obtained after isolation, but confocal microscopic analysis showed that the VEGFR2/CD31 positive signal ratio is decreased in the whole heart and in the interventricular septum. This may indirectly indicate a downregulation of this protein expression in ECs, despite elevation of the mRNA for VEGFR2. This may be due to excessive VEGFR2 trafficking and degradation with autophagy, which were previously observed in diabetic environment (Liu et al. 2012). Upregulation of VEGFR2 mRNA could be a compensatory mechanism to replace lost VEGFR2 proteins.

VEGFR2 function in ECs depends on the downstream signaling cascade. EC proliferation and differentiation, which are crucial for an angiogenic response, are mostly regulated via the PLCγ or ERK1/2 pathway (Claesson-Welsh 2016). In our experiments we observed an increased expression of mRNA for the proteins that are involved in this signaling pathway in whole heart tissue; however, the increase was not statistically significant, except for MEK and ERK1/2. On the other hand, in isolated ECs we observed a statistically significant elevation of mRNA expression for PLCγ, MEK, ERK, RAF1, and PKCβ2. In literature there are limited data describing the PLC-γ or ERK1/2 pathway in MetS hearts and the results are often contradictory. The ERK1/2 pathway may be triggered in different types of cells, and results obtained from the whole cardiac tissue may be misleading with regard to the angiogenic response. Data show that in cardiomyocytes of db/db mice with MetS there are no major changes in the expression of ERK1/2 pathway kinases (Marsh et al. 2011). Similarly MetS conditions in a porcine model do not affect ERK1/2 expression or activation (Huang et al. 2013). On the other hand, in the whole myocardium increased activation of the ERK1/2 pathway was observed in db/db mice and in Zucker Diabetic Fatty rats, which also develop MetS conditions (Chen et al. 2018; Guleria et al. 2013). Elevated levels of phosphorylated ERK1/2 may correlate with elevated oxidative stress in aortic samples from obese rats (Touati et al. 2015). Taken together, our results suggest an upregulation of the mRNA expression for ERK signaling pathway molecules in the hearts in MetS, both in cardiac tissue and in isolated ECs. However, the latter may be an effect of elevated oxidative stress in these cells (Touati et al. 2015). Another explanation is that ERK1/2 pathway upregulation is a compensatory mechanism in ECs, which try to activate angiogenic pathways, but the EC dysfunction caused by MetS environment prevents it. Nevertheless, further experiments need to be performed to support these assumptions, since our evaluations were performed only with mRNA levels and these may not correspond with protein levels or the activation of specific molecules.

AKT-signaling pathway activation results in an increased expression of the mTOR2 complex, which is an important intracellular factor that facilitates new vessel formation by regulating cell proliferation, adhesion, and motility (Simons et al. 2016). There are many contradictory studies that describe AKT signaling in MetS (Park et al. 2009; Guleria et al. 2013). In this paper, we show, that there are no changes in mRNA expression for most AKT signaling cascade molecules (SRC, FAK, PXN, VE-cadherin, AKT1, PIK3ca, eNOS, FOXO1, and PTEN), except for FOXO3a, in the whole heart tissue, although for our experiments we did not choose any specific area of the heart, which may have affected our findings. On the other hand, all of the above factors are significantly elevated in isolated ECs. In the cardiac muscle of db/db mice the AKT pathway seems to be downregulated, as demonstrated by an observed decrease in phosphorylated AKT in tissue extracts (Park et al. 2009). Moreover, this effect is not observed in cardiomyocytes, which suggests that the AKT pathway may also be affected in other cells, such as fibroblasts, immune cells, or ECs (Marsh et al. 2011). Decreased AKT phosphorylation was also observed in a diet-induced MetS rat model (Ibarra-Lara et al. 2016).

In our experiments we also observed upregulated expression of mRNA for FOXO3a, a factor that enhances apoptosis in mature ECs via downregulation of antiapoptotic inhibitory proteins and attenuates EC proliferation (Skurk et al. 2004; Potente et al. 2003), both in whole cardiac tissue and in isolated ECs. FOXO3a negatively affects angiogenesis and EC proliferation by downregulating eNOS expression (Potente et al. 2005). We did not observe downregulation of mRNA for eNOS in isolated ECs, but the expression of mRNA for the Ki67 proliferation marker (Lashen et al. 2023) was decreased in the whole cardiac tissue. This may be at least partially owing to FOXO3a mRNA upregulation, although we did not observe the difference in mRNA expression for Ki67 in isolated db/db ECs, despite increased mRNA expression for FOXO3a.

In the next step we performed an aortic ring assay, which suggests, that new sprout formation is impaired in db/db mice, which was also reported earlier (Liu et al. 2012; Li et al. 2016). We also analyzed the mRNA for VEGFR2 and EKR1/2 molecules in aortic explants, which are crucial in regulating EC proliferation and angiogenesis, and we found that only ERK1/2 mRNA was upregulated in db/db mouse aortas treated with the proangiogenic factor (VEGF_164_). Previous reports show that diabetic Akita mice exhibited a downregulation in VEGFR2 levels in an aortic ring model, but this may be owing to the fact that the VEGFR2 protein is degraded in cytosol, instead of being trafficked from endosomes to the plasma membrane, thus its mRNA levels may be unaffected (Liu et al. 2012).

### Junctional gaps are widened in microvessels in db/db cardiac muscle

Vascular permeability is controlled by three mechanisms: interactions between ECs and mural cells, transcellular transport via vesicles, and paracellular transport between ECs, which depends on the integrity of cellular junctions (Goddard and Iruela-Arispe 2013; Miyawaki-Shimizu et al. 2006). Our ultrastructural analysis of db/db mouse microvessels within some regions of the heart showed widened junctional gaps between ECs and a decreased vesicular index, which may suggest changes in microvessel permeability and/or an impaired microvessel barrier (Weis et al. 2004b; McDonald et al. 1999). VEGF downstream signaling pathway molecules, namely src kinases, as well as high levels of ROS, may affect transcellular trafficking through interaction with caveolin 1, the main protein involved in vesicle formation in ECs (Sun et al. 2009). Activation of the SRC signaling pathway results in VE-cadherin phosphorylation and endocytosis, destruction of adherents junctions, and increased endothelial permeability (Simons et al. 2016; Weis et al. 2004a). VE-cadherin may also couple with VEGFR2, and an increased phosphorylation of VE-cadherin causes uncoupling and weakening of cell-to-cell interactions and thus impairs the integrity of the vascular barrier (Shiou et al. 2019). Elevated levels of VEGF-A may additionally promote VE-cadherin phosphorylation via the VEGF-A/VEGFR/SRC axis (Weis et al. 2004b). In our work, we observed, that the expression of mRNA for VE-cadherin and VEGF-A is increased in ECs isolated from db/db mice but not in the whole heart. The VE-cadherin/CD31 ratio, analyzed with confocal microscopy, showed that VE-cadherin expression is downregulated in the left ventricle, but elevated in the right ventricle. This may indicate that different parts of the heart are affected by MetS environment in different ways. Additionally, elevated mRNA for VE-cadherin is insufficient to supplement the loss of VE-cadherin proteins.

### LEPR deficiency in db/db mouse may be a possible cause of poor angiogenic response in cardiac muscle

VEGF–VEGFR signaling is not the only angiogenic pathway in the heart, although our experiments focused only on molecules involved in this axis. It needs to be mentioned that a poor angiogenic response may be also a result of the animal model used in our experiments, since db/db mice do not have functional receptors for leptin. Leptin is known to exert vascular effects, although its cardiovascular function remains controversial, since different, often contradictory, effects of this adipokine have been reported. Leptin may be involved in the regulation of vascular tone, arterial blood pressure, and vascular relaxation via different central and peripheral mechanisms (extensively described in (Ren 2004; Mellott and Faulkner 2023)]); therefore, defective leptin signaling may lead to pathological changes within myocardium, which can ultimately contribute to HF. Leptin may enhance angiogenesis, ECs survival and migration, and tube formation in vitro (Tahergorabi and Khazaei 2015) and in vivo, i.e., in a corneal angiogenic model or in a chorioallantoic membrane model (CAM) (Bouloumie et al. 1998; Park et al. 2001; Sierra-Honigmann et al. 1998). Leptin signaling promotes tube formation through interaction with the VEGFR2 downstream signaling cascade in cultured human umbilical vein ECs (HUVECs) (Garonna et al. 2011), whereas in cancer cells, LEPR activation stimulates VEGF-A expression mainly through hypoxia-inducible factor-1 α (HIF-1α) and nuclear factor kappa-light-chain-enhancer of activated B cells (NFκB) (Gonzalez-Perez et al. 2010). LEPRs are present on ECs (Schroeter et al. 2007), but prolonged hyperleptinemia may alter LEPR expression, its distribution, or its downstream signaling cascade. Hubert et al. showed that mice fed a high-fat diet exhibit reduced LEPR expression and leptin-induced STAT3 phosphorylation (Hubert et al. 2017). Additionally, obesity may increase the levels of soluble LEPR, which limits leptin bioavailability (Hubert et al. 2017). Systemic LEPR deficiency affects all LEPR-expressing cells thus any cell-specific effects may be distorted. There are mouse models with reduced LEPR expression in ECs only, which mimic peripheral leptin resistance and show a vascular phenotype similar to that of HFD-fed WT mice (Hubert et al. 2017). On the other hand, deletion of LEPR in cardiac ECs promotes angiogenic sprouting in vitro, restores density of microvessels in cardiac muscle and reduces EC apoptosis after pressure overload (Gogiraju et al. 2019). Since db/db mice lack functional LEPR, we cannot rule out the possibility, that a poor angiogenic response in our aortic ring model was also a result of insufficient LEPR–VEGFR interactions, but this phenomenon needs further studies.

## Final conclusions and limitations of the study

Expression of mRNAs for selected proangiogenic factors and the signaling molecules involved in angiogenesis, cell survival, and vascular permeability is altered in db/db mouse ECs isolated from cardiac muscle, whereas it remains unaltered when the entire cardiac tissue is analyzed. Our studies show that isolation and analysis of ECs is crucial for evaluating cellular processes, especially in cardiac muscle, where the presence of other cell types may obscure the view. Morphological changes within the cardiac muscle, namely microvascular rarefaction and widened vascular junctional gaps, may be caused by deregulated expression of mRNA for VEGFR signaling cascade molecules, but these are preliminary observations that need further studies. Our paper has some limitations, examination of such complicated processes as EC physiology and function in tissue environment is subject to error. We were unable to show the changes in selected molecules on the protein level or evaluate the activity of proteins involved in the VEGF signaling cascade owing to the limited number of cells sorted. But we believe that our findings are important for the following reasons: first, they demonstrate new aspects of VEGF signaling in ECs only, not in the whole cardiac tissue, in the MetS environment, which are important from the point of view of clinical medicine; second, our findings may serve as a stepping stone for further research, including functional studies.

## Data Availability

No datasets were generated or analyzed during the current study.

## References

[CR1] Afshin A, Forouzanfar MH, Reitsma MB, Sur P, Estep K, Lee A, Marczak L, Mokdad AH, Moradi-Lakeh M, Naghavi M, Salama JS, Vos T, Abate KH, Abbafati C, Ahmed MB, Al-Aly Z, Alkerwi A, Al-Raddadi R, Amare AT, Amberbir A, Amegah AK, Amini E, Amrock SM, Anjana RM, Ärnlöv J, Asayesh H, Banerjee A, Barac A, Baye E, Bennett DA, Beyene AS, Biadgilign S, Biryukov S, Bjertness E, Boneya DJ, Campos-Nonato I, Carrero JJ, Cecilio P, Cercy K, Ciobanu LG, Cornaby L, Damtew SA, Dandona L, Dandona R, Dharmaratne SD, Duncan BB, Eshrati B, Esteghamati A, Feigin VL, Fernandes JC, Fürst T, Gebrehiwot TT, Gold A, Gona PN, Goto A, Habtewold TD, Hadush KT, Hafezi-Nejad N, Hay SI, Horino M, Islami F, Kamal R, Kasaeian A, Katikireddi SV, Kengne AP, Kesavachandran CN, Khader YS, Khang YH, Khubchandani J, Kim D, Kim YJ, Kinfu Y, Kosen S, Ku T, Defo BK, Kumar GA, Larson HJ, Leinsalu M, Liang X, Lim SS, Liu P, Lopez AD, Lozano R, Majeed A, Malekzadeh R, Malta DC, Mazidi M, McAlinden C, McGarvey ST, Mengistu DT, Mensah GA, Mensink GBM, Mezgebe HB, Mirrakhimov EM, Mueller UO, Noubiap JJ, Obermeyer CM, Ogbo FA, Owolabi MO, Patton GC, Pourmalek F, Qorbani M, Rafay A, Rai RK, Ranabhat CL, Reinig N, Safiri S, Salomon JA, Sanabria JR, Santos IS, Sartorius B, Sawhney M, Schmidhuber J, Schutte AE, Schmidt MI, Sepanlou SG, Shamsizadeh M, Sheikhbahaei S, Shin MJ, Shiri R, Shiue I, Roba HS, Silva DAS, Silverberg JI, Singh JA, Stranges S, Swaminathan S, Tabarés-Seisdedos R, Tadese F, Tedla BA, Tegegne BS, Terkawi AS, Thakur JS, Tonelli M, Topor-Madry R, Tyrovolas S, Ukwaja KN, Uthman OA, Vaezghasemi M, Vasankari T, Vlassov VV, Vollset SE, Weiderpass E, Werdecker A, Wesana J, Westerman R, Yano Y, Yonemoto N, Yonga G, Zaidi Z, Zenebe ZM, Zipkin B, Murray CJL (2017) Health effects of overweight and obesity in 195 countries over 25 years. N Engl J Med 377(1):13–27. 10.1056/NEJMoa1614362 28604169 10.1056/NEJMoa1614362 PMC5477817

[CR2] Alex L, Russo I, Holoborodko V, Frangogiannis NG (2018) Characterization of a mouse model of obesity-related fibrotic cardiomyopathy that recapitulates features of human heart failure with preserved ejection fraction. Am J Physiol Heart Circ Physiol 315(4):H934–H949. 10.1152/ajpheart.00238.2018 30004258 10.1152/ajpheart.00238.2018 PMC6230908

[CR3] Bartkowiak K, Bartkowiak M, Jankowska-Steifer E, Ratajska A, Kujawa M, Aniolek O, Niderla-Bielinska J (2024) Metabolic syndrome and cardiac vessel remodeling associated with vessel rarefaction: a possible underlying mechanism may result from a poor angiogenic response to altered VEGF signaling pathways. J Vasc Res. 10.1159/000538361 38615659 10.1159/000538361

[CR4] Berger C, Kloting N (2021) Leptin receptor compound heterozygosity in humans and animal models. Int J Mol Sci. 10.3390/ijms22094475 33922961 10.3390/ijms22094475 PMC8123313

[CR5] Bouloumie A, Drexler HC, Lafontan M, Busse R (1998) Leptin, the product of Ob gene, promotes angiogenesis. Circ Res 83(10):1059–1066. 10.1161/01.res.83.10.1059 9815153 10.1161/01.res.83.10.1059

[CR6] Broderick TL, Parrott CR, Wang D, Jankowski M, Gutkowska J (2012) Expression of cardiac GATA4 and downstream genes after exercise training in the db/db mouse. Pathophysiology 19(3):193–203. 10.1016/j.pathophys.2012.06.001 22809789 10.1016/j.pathophys.2012.06.001

[CR7] Broderick TL, Sennott JM, Gutkowska J, Jankowski M (2019) Anti-inflammatory and angiogenic effects of exercise training in cardiac muscle of diabetic mice. Diabetes Metab Syndr Obes 12:565–573. 10.2147/dmso.S197127 31118719 10.2147/DMSO.S197127 PMC6499146

[CR8] Chen JX, Zeng H, Reese J, Aschner JL, Meyrick B (2012) Overexpression of angiopoietin-2 impairs myocardial angiogenesis and exacerbates cardiac fibrosis in the diabetic db/db mouse model. Am J Physiol Heart Circ Physiol 302(4):H1003-1012. 10.1152/ajpheart.00866.2011 22180648 10.1152/ajpheart.00866.2011 PMC3322731

[CR9] Chen W, Sun Q, Ju J, Chen W, Zhao X, Zhang Y, Yang Y (2018) Effect of Astragalus polysaccharides on cardiac dysfunction in db/db mice with respect to oxidant stress. Biomed Res Int 2018:8359013. 10.1155/2018/8359013 30581869 10.1155/2018/8359013 PMC6276493

[CR10] Cheng HS, Perez-Cremades D, Zhuang R, Jamaiyar A, Wu W, Chen J, Tzani A, Stone L, Plutzky J, Ryan TE, Goodney PP, Creager MA, Sabatine MS, Bonaca MP, Feinberg MW (2023) Impaired angiogenesis in diabetic critical limb ischemia is mediated by a miR-130b/INHBA signaling axis. JCI Insight. 10.1172/jci.insight.163041 37097749 10.1172/jci.insight.163041 PMC10322685

[CR11] Chou E, Suzuma I, Way KJ, Opland D, Clermont AC, Naruse K, Suzuma K, Bowling NL, Vlahos CJ, Aiello LP, King GL (2002) Decreased cardiac expression of vascular endothelial growth factor and its receptors in insulin-resistant and diabetic States: a possible explanation for impaired collateral formation in cardiac tissue. Circulation 105(3):373–379. 10.1161/hc0302.102143 11804995 10.1161/hc0302.102143

[CR12] Claesson-Welsh L (2016) VEGF receptor signal transduction—a brief update. Vascul Pharmacol 86:14–17. 10.1016/j.vph.2016.05.011 27268035 10.1016/j.vph.2016.05.011

[CR13] Dham D, Roy B, Gowda A, Pan G, Sridhar A, Zeng X, Thandavarayan RA, Palaniyandi SS (2021) 4-Hydroxy-2-nonenal, a lipid peroxidation product, as a biomarker in diabetes and its complications: challenges and opportunities. Free Radic Res 55(5):547–561. 10.1080/10715762.2020.1866756 33336611 10.1080/10715762.2020.1866756 PMC8260649

[CR14] Dobrzynska MM, Gajowik A, Radzikowska J, Tyrkiel EJ, Jankowska-Steifer EA (2015) Male-mediated F1 effects in mice exposed to bisphenol A, either alone or in combination with X-irradiation. Mutat Res Genet Toxicol Environ Mutagen 789–790:36–45. 10.1016/j.mrgentox.2015.06.015 26232256 10.1016/j.mrgentox.2015.06.015

[CR15] Fahed G, Aoun L, Bou Zerdan M, Allam S, Bou Zerdan M, Bouferraa Y, Assi HI (2022) Metabolic syndrome: updates on pathophysiology and management in 2021. Int J Mol Sci. 10.3390/ijms23020786 35054972 10.3390/ijms23020786 PMC8775991

[CR16] Farooqi IS, Wangensteen T, Collins S, Kimber W, Matarese G, Keogh JM, Lank E, Bottomley B, Lopez-Fernandez J, Ferraz-Amaro I, Dattani MT, Ercan O, Myhre AG, Retterstol L, Stanhope R, Edge JA, McKenzie S, Lessan N, Ghodsi M, De Rosa V, Perna F, Fontana S, Barroso I, Undlien DE, O’Rahilly S (2007) Clinical and molecular genetic spectrum of congenital deficiency of the leptin receptor. N Engl J Med 356(3):237–247. 10.1056/NEJMoa063988 17229951 10.1056/NEJMoa063988 PMC2670197

[CR17] Fellmann L, Nascimento AR, Tibiriça E, Bousquet P (2013) Murine models for pharmacological studies of the metabolic syndrome. Pharmacol Ther 137(3):331–340. 10.1016/j.pharmthera.2012.11.004 23178510 10.1016/j.pharmthera.2012.11.004

[CR18] Flaht-Zabost A, Gula G, Ciszek B, Czarnowska E, Jankowska-Steifer E, Madej M, Niderla-Bielinska J, Radomska-Lesniewska D, Ratajska A (2014) Cardiac mouse lymphatics: developmental and anatomical update. Anat Rec 297(6):1115–1130. 10.1002/ar.22912 10.1002/ar.22912 24700724

[CR19] Garonna E, Botham KM, Birdsey GM, Randi AM, Gonzalez-Perez RR, Wheeler-Jones CP (2011) Vascular endothelial growth factor receptor-2 couples cyclo-oxygenase-2 with pro-angiogenic actions of leptin on human endothelial cells. PLoS ONE 6(4):e18823. 10.1371/journal.pone.0018823 21533119 10.1371/journal.pone.0018823 PMC3078934

[CR20] Goddard LM, Iruela-Arispe ML (2013) Cellular and molecular regulation of vascular permeability. Thromb Haemost 109(3):407–415. 10.1160/TH12-09-0678 23389236 10.1160/TH12-09-0678 PMC3786592

[CR21] Gogiraju R, Hubert A, Fahrer J, Straub BK, Brandt M, Wenzel P, Munzel T, Konstantinides S, Hasenfuss G, Schafer K (2019) Endothelial leptin receptor deletion promotes cardiac autophagy and angiogenesis following pressure overload by suppressing Akt/mTOR signaling. Circ Heart Fail 12(1):e005622. 10.1161/CIRCHEARTFAILURE.118.005622 30621510 10.1161/CIRCHEARTFAILURE.118.005622

[CR22] Gogiraju R, Witzler C, Shahneh F, Hubert A, Renner L, Bochenek ML, Zifkos K, Becker C, Thati M, Schafer K (2023) Deletion of endothelial leptin receptors in mice promotes diet-induced obesity. Sci Rep 13(1):8276. 10.1038/s41598-023-35281-7 37217565 10.1038/s41598-023-35281-7 PMC10203363

[CR23] Gonzalez-Perez RR, Xu Y, Guo S, Watters A, Zhou W, Leibovich SJ (2010) Leptin upregulates VEGF in breast cancer via canonic and non-canonical signalling pathways and NFkappaB/HIF-1alpha activation. Cell Signal 22(9):1350–1362. 10.1016/j.cellsig.2010.05.003 20466060 10.1016/j.cellsig.2010.05.003 PMC2928711

[CR24] Guleria RS, Singh AB, Nizamutdinova IT, Souslova T, Mohammad AA, Kendall JA Jr, Baker KM, Pan J (2013) Activation of retinoid receptor-mediated signaling ameliorates diabetes-induced cardiac dysfunction in Zucker diabetic rats. J Mol Cell Cardiol 57:106–118. 10.1016/j.yjmcc.2013.01.017 23395853 10.1016/j.yjmcc.2013.01.017 PMC3594065

[CR25] Huang JV, Lu L, Ye S, Bergman BC, Sparagna GC, Sarraf M, Reusch JE, Greyson CR, Schwartz GG (2013) Impaired contractile recovery after low-flow myocardial ischemia in a porcine model of metabolic syndrome. Am J Physiol Heart Circ Physiol 304(6):H861-873. 10.1152/ajpheart.00535.2012 23335793 10.1152/ajpheart.00535.2012 PMC3602770

[CR26] Hubert A, Bochenek ML, Schutz E, Gogiraju R, Munzel T, Schafer K (2017) Selective deletion of leptin signaling in endothelial cells enhances neointima formation and phenocopies the vascular effects of diet-induced obesity in mice. Arterioscler Thromb Vasc Biol 37(9):1683–1697. 10.1161/ATVBAHA.117.309798 28705795 10.1161/ATVBAHA.117.309798

[CR27] Ibarra-Lara L, Sanchez-Aguilar M, Sanchez-Mendoza A, Del Valle-Mondragon L, Soria-Castro E, Carreon-Torres E, Diaz-Diaz E, Vazquez-Meza H, Guarner-Lans V, Rubio-Ruiz ME (2016) Fenofibrate therapy restores antioxidant protection and improves myocardial insulin resistance in a rat model of metabolic syndrome and myocardial ischemia: the role of angiotensin II. Molecules. 10.3390/molecules22010031 28036029 10.3390/molecules22010031 PMC6155612

[CR28] Karaman S, Leppanen VM, Alitalo K (2018) Vascular endothelial growth factor signaling in development and disease. Development. 10.1242/dev.151019 30030240 10.1242/dev.151019

[CR29] Krüger-Genge A, Blocki A, Franke RP, Jung F (2019) Vascular endothelial cell biology: an update. Int J Mol Sci. 10.3390/ijms20184411 31500313 10.3390/ijms20184411 PMC6769656

[CR30] Lashen AG, Toss MS, Ghannam SF, Makhlouf S, Green A, Mongan NP, Rakha E (2023) Expression, assessment and significance of Ki67 expression in breast cancer: an update. J Clin Pathol. 10.1136/jcp-2022-208731 36813558 10.1136/jcp-2022-208731

[CR31] Leifheit-Nestler M, Wagner NM, Gogiraju R, Didie M, Konstantinides S, Hasenfuss G, Schafer K (2013) Importance of leptin signaling and signal transducer and activator of transcription-3 activation in mediating the cardiac hypertrophy associated with obesity. J Transl Med 11:170. 10.1186/1479-5876-11-170 23841921 10.1186/1479-5876-11-170 PMC3717024

[CR32] Li R, Xie J, Wu H, Li G, Chen J, Chen Q, Wang L, Xu B (2016) Syndecan-4 shedding impairs macrovascular angiogenesis in diabetes mellitus. Biochem Biophys Res Commun 474(1):15–21. 10.1016/j.bbrc.2016.03.112 27018253 10.1016/j.bbrc.2016.03.112

[CR33] Litviňuková M, Talavera-López C, Maatz H, Reichart D, Worth CL, Lindberg EL, Kanda M, Polanski K, Heinig M, Lee M, Nadelmann ER, Roberts K, Tuck L, Fasouli ES, DeLaughter DM, McDonough B, Wakimoto H, Gorham JM, Samari S, Mahbubani KT, Saeb-Parsy K, Patone G, Boyle JJ, Zhang H, Zhang H, Viveiros A, Oudit GY, Bayraktar OA, Seidman JG, Seidman CE, Noseda M, Hubner N, Teichmann SA (2020) Cells of the adult human heart. Nature 588(7838):466–472. 10.1038/s41586-020-2797-4 32971526 10.1038/s41586-020-2797-4 PMC7681775

[CR34] Liu H, Yu S, Zhang H, Xu J (2012) Angiogenesis impairment in diabetes: role of methylglyoxal-induced receptor for advanced glycation endproducts, autophagy and vascular endothelial growth factor receptor 2. PLoS ONE 7(10):e46720. 10.1371/journal.pone.0046720 23056421 10.1371/journal.pone.0046720 PMC3463541

[CR35] Liu IF, Lin TC, Wang SC, Yen CH, Li CY, Kuo HF, Hsieh CC, Chang CY, Chang CR, Chen YH, Liu YR, Lee TY, Huang CY, Hsu CH, Lin SJ, Liu PL (2023) Long-term administration of Western diet induced metabolic syndrome in mice and causes cardiac microvascular dysfunction, cardiomyocyte mitochondrial damage, and cardiac remodeling involving caveolae and caveolin-1 expression. Biol Direct 18(1):9. 10.1186/s13062-023-00363-z 36879344 10.1186/s13062-023-00363-z PMC9987103

[CR36] Marsh SA, Dell’Italia LJ, Chatham JC (2011) Activation of the hexosamine biosynthesis pathway and protein O-GlcNAcylation modulate hypertrophic and cell signaling pathways in cardiomyocytes from diabetic mice. Amino Acids 40(3):819–828. 10.1007/s00726-010-0699-8 20676904 10.1007/s00726-010-0699-8 PMC3025273

[CR37] Mazidi M, Rezaie P, Kengne AP, Stathopoulou MG, Azimi-Nezhad M, Siest S (2017) VEGF, the underlying factor for metabolic syndrome; fact or fiction? Diabetes Metab Syndr 11(Suppl 1):S61-s64. 10.1016/j.dsx.2016.12.004 28040466 10.1016/j.dsx.2016.12.004

[CR38] McDonald DM, Thurston G, Baluk P (1999) Endothelial gaps as sites for plasma leakage in inflammation. Microcirculation 6(1):7–22 10100186

[CR39] Meldrum DR, Morris MA, Gambone JC (2017) Obesity pandemic: causes, consequences, and solutions-but do we have the will? Fertil Steril 107(4):833–839. 10.1016/j.fertnstert.2017.02.104 28292617 10.1016/j.fertnstert.2017.02.104

[CR40] Mellott E, Faulkner JL (2023) Mechanisms of leptin-induced endothelial dysfunction. Curr Opin Nephrol Hypertens 32(2):118–123. 10.1097/MNH.0000000000000867 36598435 10.1097/MNH.0000000000000867 PMC9870925

[CR41] Miyawaki-Shimizu K, Predescu D, Shimizu J, Broman M, Predescu S, Malik AB (2006) siRNA-induced caveolin-1 knockdown in mice increases lung vascular permeability via the junctional pathway. Am J Physiol Lung Cell Mol Physiol 290(2):L405-413. 10.1152/ajplung.00292.2005 16183667 10.1152/ajplung.00292.2005

[CR42] Mohammed SF, Hussain S, Mirzoyev SA, Edwards WD, Maleszewski JJ, Redfield MM (2015) Coronary microvascular rarefaction and myocardial fibrosis in heart failure with preserved ejection fraction. Circulation 131(6):550–559. 10.1161/circulationaha.114.009625 25552356 10.1161/CIRCULATIONAHA.114.009625 PMC4324362

[CR43] Niderla-Bielinska J, Sciezynska A, Moskalik A, Jankowska-Steifer E, Bartkowiak K, Bartkowiak M, Kiernozek E, Podgorska A, Ciszek B, Majchrzak B, Ratajska A (2021) A comprehensive miRNome analysis of macrophages isolated from db/db mice and selected miRNAs involved in metabolic syndrome-associated cardiac remodeling. Int J Mol Sci. 10.3390/ijms22042197 33672153 10.3390/ijms22042197 PMC7926522

[CR44] Ortega FB, Lavie CJ, Blair SN (2016) Obesity and cardiovascular disease. Circ Res 118(11):1752–1770. 10.1161/circresaha.115.306883 27230640 10.1161/CIRCRESAHA.115.306883

[CR45] Park HY, Kwon HM, Lim HJ, Hong BK, Lee JY, Park BE, Jang Y, Cho SY, Kim HS (2001) Potential role of leptin in angiogenesis: leptin induces endothelial cell proliferation and expression of matrix metalloproteinases in vivo and in vitro. Exp Mol Med 33(2):95–102. 10.1038/emm.2001.17 11460888 10.1038/emm.2001.17

[CR46] Park CW, Kim HW, Lim JH, Yoo KD, Chung S, Shin SJ, Chung HW, Lee SJ, Chae CB, Kim YS, Chang YS (2009) Vascular endothelial growth factor inhibition by dRK6 causes endothelial apoptosis, fibrosis, and inflammation in the heart via the Akt/eNOS axis in db/db mice. Diabetes 58(11):2666–2676. 10.2337/db09-0136 19675133 10.2337/db09-0136 PMC2768173

[CR47] Peach CJ, Mignone VW, Arruda MA, Alcobia DC, Hill SJ, Kilpatrick LE, Woolard J (2018) Molecular pharmacology of VEGF-A isoforms: binding and signalling at VEGFR2. Int J Mol Sci. 10.3390/ijms19041264 29690653 10.3390/ijms19041264 PMC5979509

[CR48] Potente M, Fisslthaler B, Busse R, Fleming I (2003) 11,12-Epoxyeicosatrienoic acid-induced inhibition of FOXO factors promotes endothelial proliferation by down-regulating p27Kip1. J Biol Chem 278(32):29619–29625. 10.1074/jbc.M305385200 12773534 10.1074/jbc.M305385200

[CR49] Potente M, Urbich C, Sasaki K, Hofmann WK, Heeschen C, Aicher A, Kollipara R, DePinho RA, Zeiher AM, Dimmeler S (2005) Involvement of Foxo transcription factors in angiogenesis and postnatal neovascularization. J Clin Invest 115(9):2382–2392. 10.1172/JCI23126 16100571 10.1172/JCI23126 PMC1184037

[CR50] Purwowiyoto SL, Prawara AS (2021) Metabolic syndrome and heart failure: mechanism and management. Med Pharm Rep 94(1):15–21. 10.15386/mpr-1884 33629043 10.15386/mpr-1884 PMC7880077

[CR51] Raman P, Khanal S (2021) Leptin in atherosclerosis: focus on macrophages, endothelial and smooth muscle cells. Int J Mol Sci. 10.3390/ijms22115446 34064112 10.3390/ijms22115446 PMC8196747

[CR52] Rawal S, Munasinghe PE, Shindikar A, Paulin J, Cameron V, Manning P, Williams MJ, Jones GT, Bunton R, Galvin I, Katare R (2017) Down-regulation of proangiogenic microRNA-126 and microRNA-132 are early modulators of diabetic cardiac microangiopathy. Cardiovasc Res 113(1):90–101. 10.1093/cvr/cvw235 28065883 10.1093/cvr/cvw235

[CR53] Ren J (2004) Leptin and hyperleptinemia—from friend to foe for cardiovascular function. J Endocrinol 181(1):1–10. 10.1677/joe.0.1810001 15072562 10.1677/joe.0.1810001

[CR54] Ren J, Zhu BH, Relling DP, Esberg LB, Ceylan-Isik AF (2008) High-fat diet-induced obesity leads to resistance to leptin-induced cardiomyocyte contractile response. Obesity (Silver Spring) 16(11):2417–2423. 10.1038/oby.2008.381 18719678 10.1038/oby.2008.381

[CR55] Roy B, Pan G, Giri S, Thandavarayan RA, Palaniyandi SS (2022) Aldehyde dehydrogenase 2 augments adiponectin signaling in coronary angiogenesis in HFpEF associated with diabetes. FASEB J 36(8):e22440. 10.1096/fj.202200498R 35815932 10.1096/fj.202200498R

[CR56] Saltiel AR, Olefsky JM (2017) Inflammatory mechanisms linking obesity and metabolic disease. J Clin Invest 127(1):1–4. 10.1172/jci92035 28045402 10.1172/JCI92035 PMC5199709

[CR57] Salvatore T, Galiero R, Caturano A, Vetrano E, Loffredo G, Rinaldi L, Catalini C, Gjeloshi K, Albanese G, Di Martino A, Docimo G, Sardu C, Marfella R, Sasso FC (2022) Coronary microvascular dysfunction in diabetes mellitus: pathogenetic mechanisms and potential therapeutic options. Biomedicines. 10.3390/biomedicines10092274 36140374 10.3390/biomedicines10092274 PMC9496134

[CR58] Schroeter MR, Schneiderman J, Schumann B, Gluckermann R, Grimmas P, Buchwald AB, Tirilomis T, Schondube FA, Konstantinides SV, Schafer K (2007) Expression of the leptin receptor in different types of vascular lesions. Histochem Cell Biol 128(4):323–333. 10.1007/s00418-007-0319-1 17680264 10.1007/s00418-007-0319-1

[CR59] Schwartz MW, Peskind E, Raskind M, Boyko EJ, Porte D Jr (1996) Cerebrospinal fluid leptin levels: relationship to plasma levels and to adiposity in humans. Nat Med 2(5):589–593. 10.1038/nm0596-589 8616722 10.1038/nm0596-589

[CR60] Sherling DH, Perumareddi P, Hennekens CH (2017) Metabolic syndrome. J Cardiovasc Pharmacol Ther 22(4):365–367. 10.1177/1074248416686187 28587579 10.1177/1074248416686187

[CR61] Shiou YL, Lin HT, Ke LY, Wu BN, Shin SJ, Chen CH, Tsai WC, Chu CS, Lee HC (2019) Very low-density lipoproteins of metabolic syndrome modulates STIM1, suppresses store-operated calcium entry, and deranges myofilament proteins in atrial myocytes. J Clin Med. 10.3390/jcm8060881 31226824 10.3390/jcm8060881 PMC6617489

[CR62] Sierra-Honigmann MR, Nath AK, Murakami C, Garcia-Cardena G, Papapetropoulos A, Sessa WC, Madge LA, Schechner JS, Schwabb MB, Polverini PJ, Flores-Riveros JR (1998) Biological action of leptin as an angiogenic factor. Science 281(5383):1683–1686. 10.1126/science.281.5383.1683 9733517 10.1126/science.281.5383.1683

[CR63] Simmonds SJ, Cuijpers I, Heymans S, Jones EAV (2020) Cellular and molecular differences between HFpEF and HFrEF: a step ahead in an improved pathological understanding. Cells. 10.3390/cells9010242 31963679 10.3390/cells9010242 PMC7016826

[CR64] Simons M, Gordon E, Claesson-Welsh L (2016) Mechanisms and regulation of endothelial VEGF receptor signalling. Nat Rev Mol Cell Biol 17(10):611–625. 10.1038/nrm.2016.87 27461391 10.1038/nrm.2016.87

[CR65] Skurk C, Maatz H, Kim HS, Yang J, Abid MR, Aird WC, Walsh K (2004) The Akt-regulated forkhead transcription factor FOXO3a controls endothelial cell viability through modulation of the caspase-8 inhibitor FLIP. J Biol Chem 279(2):1513–1525. 10.1074/jbc.M304736200 14551207 10.1074/jbc.M304736200

[CR66] Sun Y, Hu G, Zhang X, Minshall RD (2009) Phosphorylation of caveolin-1 regulates oxidant-induced pulmonary vascular permeability via paracellular and transcellular pathways. Circ Res. 10.1161/CIRCRESAHA.109.201673 19713536 10.1161/CIRCRESAHA.109.201673 PMC2776728

[CR67] Tahergorabi Z, Khazaei M (2015) Leptin and its cardiovascular effects: focus on angiogenesis. Adv Biomed Res 4:79. 10.4103/2277-9175.156526 26015905 10.4103/2277-9175.156526 PMC4434486

[CR68] Taqueti VR, Solomon SD, Shah AM, Desai AS, Groarke JD, Osborne MT, Hainer J, Bibbo CF, Dorbala S, Blankstein R, Di Carli MF (2018) Coronary microvascular dysfunction and future risk of heart failure with preserved ejection fraction. Eur Heart J 39(10):840–849. 10.1093/eurheartj/ehx721 29293969 10.1093/eurheartj/ehx721 PMC5939665

[CR69] Touati S, Montezano AC, Meziri F, Riva C, Touyz RM, Laurant P (2015) Exercise training protects against atherosclerotic risk factors through vascular NADPH oxidase, extracellular signal-regulated kinase 1/2 and stress-activated protein kinase/c-Jun N-terminal kinase downregulation in obese rats. Clin Exp Pharmacol Physiol 42(2):179–185. 10.1111/1440-1681.12338 25399833 10.1111/1440-1681.12338

[CR70] Veitch S, Njock MS, Chandy M, Siraj MA, Chi L, Mak H, Yu K, Rathnakumar K, Perez-Romero CA, Chen Z, Alibhai FJ, Gustafson D, Raju S, Wu R, Zarrin Khat D, Wang Y, Caballero A, Meagher P, Lau E, Pepic L, Cheng HS, Galant NJ, Howe KL, Li RK, Connelly KA, Husain M, Delgado-Olguin P, Fish JE (2022) MiR-30 promotes fatty acid beta-oxidation and endothelial cell dysfunction and is a circulating biomarker of coronary microvascular dysfunction in pre-clinical models of diabetes. Cardiovasc Diabetol 21(1):31. 10.1186/s12933-022-01458-z 35209901 10.1186/s12933-022-01458-z PMC8876371

[CR71] Wauman J, Tavernier J (2011) Leptin receptor signaling: pathways to leptin resistance. Front Biosci 16(7):2771–2793. 10.2741/3885 10.2741/3885 21622208

[CR72] Weis S, Cui J, Barnes L, Cheresh D (2004a) Endothelial barrier disruption by VEGF-mediated Src activity potentiates tumor cell extravasation and metastasis. J Cell Biol 167(2):223–229. 10.1083/jcb.200408130 15504909 10.1083/jcb.200408130 PMC2172541

[CR73] Weis S, Shintani S, Weber A, Kirchmair R, Wood M, Cravens A, McSharry H, Iwakura A, Yoon YS, Himes N, Burstein D, Doukas J, Soll R, Losordo D, Cheresh D (2004b) Src blockade stabilizes a Flk/cadherin complex, reducing edema and tissue injury following myocardial infarction. J Clin Invest 113(6):885–894. 10.1172/JCI20702 15067321 10.1172/JCI20702 PMC362122

[CR74] Zafar MI, Mills K, Ye X, Blakely B, Min J, Kong W, Zhang N, Gou L, Regmi A, Hu SQ, Zheng J, Chen LL (2018) Association between the expression of vascular endothelial growth factors and metabolic syndrome or its components: a systematic review and meta-analysis. Diabetol Metab Syndr 10:62. 10.1186/s13098-018-0363-0 30087698 10.1186/s13098-018-0363-0 PMC6076391

